# Single-leg hop distance normalized to body height is associated with the return to sports after anterior cruciate ligament reconstruction

**DOI:** 10.1186/s40634-021-00344-z

**Published:** 2021-04-02

**Authors:** Shunsuke Ohji, Junya Aizawa, Kenji Hirohata, Takehiro Ohmi, Sho Mitomo, Tetsuya Jinno, Hideyuki Koga, Kazuyoshi Yagishita

**Affiliations:** 1 Clinical Center for Sports Medicine and Sports Dentistry, Tokyo Medical and Dental University, 1-5-45 Yushima, Bunkyo-ku, 113-8519 Tokyo Japan; 2 Department of Physical Therapy, Juntendo University, 3-2-12 Hongo, Bunkyo-ku, 113-0033 Tokyo Japan; 3 Department of Orthopaedic Surgery, Dokkyo Medical University Saitama Medical Center, 2-1-50 Minamikoshigaya, Koshigaya-shi, 343-8555 Saitama Japan; 4 Department of Joint Surgery and Sports Medicine, Tokyo Medical and Dental University, 1-5-45 Yushima, Bunkyo-ku, 113-8519 Tokyo Japan

**Keywords:** Anterior cruciate ligament, Anterior cruciate ligament reconstruction, Body height, Hopping, Return to play

## Abstract

**Purpose:**

To investigate the relationship between single-leg hop distance (SLHD), normalized body height, and return-to-sports (RTS) status after anterior cruciate ligament reconstruction (ACLR) and to identify the cut-off value for SLHD on the operated side.

**Methods:**

Seventy-three patients after primary ACLR (median 13.5 months) participated in this cross-sectional study. Participants were divided into ‘‘Yes-RTS’’ (YRTS) or ‘‘No-RTS’’ (NRTS) groups based on a self-reported questionnaire. SLHD was measured, and the limb symmetry index (LSI) and SLHD (%body height) were calculated. A minimum *p-*value approach was used to calculate the SLHD cut-off points (%body height) on the operated side that were strongly associated with the RTS status. Logistic regression analysis was used to analyse the association between RTS status and SLHD cut-off point (%body height). Isokinetic strength and Tampa scale for kinesiophobia (TSK) were measured as covariates.

**Results:**

Among 73 patients, 43 (59%) were assigned to the YRTS and 30 (41%) to the NRTS group. The 70% body height cut-off point for SLHD on the operated side was most strongly associated with RTS status. In a logistic regression analysis including other covariates, SLHD (%body height) < 70% and TSK were negatively associated with RTS status. Except for two participants, the LSI of the SLHD exceeded 90% and there was no significant association between the LSI of the SLHD and RTS status.

**Conclusion:**

Even after improvement in the LSI of the SLHD, planning rehabilitation with the goal of achieving SLHD over 70% body height may be important for supporting RTS after ACLR.

**Level of evidence:**

Cross-sectional study, Level IV

## Introduction

Many athletes with anterior cruciate ligament (ACL) injury undergo ACL reconstruction (ACLR) with the expectation of a return to sport (RTS) at the same level of competition as before the injury [11]. However, a meta-analysis examining RTS rates showed that approximately 40% of athletes were unable to RTS at the same level as before the injury [3].

Several previous studies have shown that post-ACLR patients unable to RTS at the same level as before the injury had a smaller limb symmetry index (LSI) in the single-leg hop distance (SLHD) than those able to RTS [4, 16, 17, 34, 35]. However, it has also been reported [21] that the LSI of the SLHD was not associated with RTS status; thus, there is no consensus view. This could be because the LSI of the SLHD approaches 100% faster than other physical function variables [6, 26]. In addition, the LSI of the SLHD can be improved by a functional decline on the unoperated side [31, 36]. For these reasons, it is necessary to focus on variables other than the LSI when assessing the SLHD.

In a cohort study examining the association between pre-season functional testing and in-season lower extremity injury in healthy athletes, injury rates were increased in those with an SLHD normalized to body height < 64‒70%, in addition to an SLHD LSI ≤ 90% [8, 9]. These findings suggest that SLHD normalized to body height can be used as a performance variable as well as an asymmetry assessment and may be related to RTS status. However, no previous study has clarified the relationship between RTS status and SLHD normalized to body height in post-ACLR patients.

Therefore, the purpose of this study was to investigate the relationship between SLHD normalized to body height and the LSI of the SLHD and RTS status after ACLR. In this study, we hypothesised that SLHD normalized to body height would be more strongly related to RTS status than the LSI of the SLHD. The present study further aimed to identify the SLHD cut-off (normalized to body height) and to present data useful for planning rehabilitation and training.

## Materials and methods

### Participants

Participants who had undergone primary ACLR between April 2012 and February 2020 were included if they met the following criteria: (1) aged 16‒45 years at the time of measurement; (2) participation in sports with a modified Tegner activity scale score [12] ≥ 5 before ACL injury; (3) time from ACLR > 8 months; (4) participation in the sport approved by their physicians; and (5) the intention to RTS had been indicated before surgery.

### Surgical technique and postoperative rehabilitation

The autograft sources were hamstrings (semitendinosus) and bone‒patellar tendon‒bone (BTB). Surgery using hamstrings was performed with an anatomical double-bundle reconstruction. If the semitendinosus alone was insufficient as a graft tendon, the gracilis was added. The surgery technique and postoperative rehabilitation protocol were based on previous research [29]. Range of motion and muscle isometric contraction exercises were initiated three days after surgery. A straight-position knee-joint immobiliser (Knee brace, ALCARE Co., Ltd., Tokyo, Japan) and crutches were initially used and then gradually phased out from four weeks after ACLR. Jogging started three months after ACLR, and the running speed gradually increased. Sports participation was allowed when the following were achieved: at least six months had passed after ACLR; the LSI on the SLHD exceeded 90%; and sufficient knee strength recovery had been attained (i.e. LSI of isokinetic extension and flexion torque > 85%, measured with an isokinetic dynamometer [BIODEX System 4, BIODEX Medical Inc., Shirley, NY] at 60°/s and 180°/s). Participants who underwent repair of the middle-posterior segment of the meniscus were prohibited from performing deep squatting to more than 90° until three months after ACLR.

### Patient characteristics

This was a cross-sectional study conducted in a single centre. Demographic information and surgical information were collected from medical records. Participants' height, weight, knee strength, SLHD, kinesiophobia, and RTS status were measured on the same day. The demographic characteristics are summarised in Table 1.

**Table 1:** Demographic variables distributions in participants

		RTS status			
	Total (*n* = 73)	YRTS (*n* = 43)	NRTS (*n* = 30)	*P*-value	Effect size
Age, y^a^	21.0 (6.0)	21.9 (4.3)	22.0 (10.3)	0.108	-0.19
Sex (female/male)	31/42	21/22	10/20	0.187	0.15
Height, cm^a^	167.0 (0.2)	166.0 (14.0)	170.3 (16.4)	0.077	-0.21
Body weight, kg^a^	63.5 (21.0)	60.0 (17.0)	70.0 (21.3)	0.045	-0.24
BMI, kg/m^2a^	22.5 (3.8)	22.5 (3.0)	23.4 (5.1)	0.148	-0.17
Days from injury to ACLR^a^	70.5 (60.0)	73.5 (88.8)	69.5 (51.8)	0.982	0.00
Months from surgery to ACLR^a^	13.5 (13.0)	14.5 (14.3)	13.0 (4.5)	0.253	-0.13
Graft type (HT/BTB)	67/6	39/4	28/2	1.000	-0.04
Meniscus repair (yes/no)	54/19	32/11	22/8	0.917	0.01
TSK score	33.4 ± 6.2	31.8 ± 6.2	35.8 ± 5.4	0.006	0.68
Pre-injury Tegner activity scale	7.8 ± 1.2	7.8 ± 1.2	7.7 ± 1.3	0.820	0.01

*RTS* return-to-sports, *YRTS* yes‒return-to-sports, *NRTS* no‒return-to-sports, *BMI* body mass index, *ACLR* anterior cruciate ligament reconstruction, *HT* hamstrings, *BTB* bone‒patellar tendon‒bone, *TSK* Tampa scale for kinesiophobia

^a^median (interquartile range)

Ethical approval was obtained from the Ethics Committee (approval number: M2016-197). All participants provided written informed consent prior to participation.

### Knee strength

Knee extension and flexion strength were assessed using an isokinetic dynamometer. Isokinetic torque was assessed at angular velocities of 60°/s and 180°/s (Ext 60 and Ext 180, Flex 60, and Flex 180, respectively). These angular velocities have commonly been used in previous studies [24, 39]. The peak torque was expressed in Newton meter (Nm). All participants completed ≥ 2 practice repetitions to become familiar with the task, followed by six maximum repetitions. The LSIs of peak torques were calculated as the ratio of the peak torque on the operated side to the peak torque on the unoperated side. High intra-rater reliability (intraclass correlation coefficient (ICC) ranged between 0.82 and 0.97) was established using the same method [7].

### SLHD

The SLHD was measured based on previous research [7]. Participants stood on a single leg behind a line representing the starting line and, from this position, hopped as far as possible, landing on the same leg. Arm movement during jump landing was not restricted. Each participant completed ≥ 3 practice jumps to become familiar with the task, which was followed by two successful trials. The test was considered successful if the landing was stable. An unsuccessful hop was classified as any of the following: additional hop on landing, landing with an early touchdown of the contralateral limb, and/or loss of balance. The distance from the starting line to the point where the back of the participant’s heel hit the ground upon completing the single hop was recorded. The maximum distance of the two trials was used for analysis. The LSI and distance-normalized body height (%body height) were calculated. High intra-rater reliability (ICC ranged between 0.88 and 0.97) was previously established using the same method [7].

### Kinesiophobia

In the present study, we measured kinesiophobia (fear of re-injury and fear of movement) as a psychological variable that could be associated with RTS after ACLR [28]. Kinesiophobia was measured using the Japanese version of the Tampa Scale for Kinesiophobia (TSK) [15]. The TSK is a 17-item questionnaire scored on a four-point Likert scale. Total scores ranged from 17 to 68, with higher scores indicating greater kinesiophobia. The TSK has been reported to have good internal consistency [38].

### RTS status

RTS status was defined by two self-reported questions. The dichotomous (yes/no) question was, “Have you returned to the same level of competition as before your ACL injury?” This question was mostly used in previous studies [2, 4, 20, 22]. The continuous-response (0%–100%) question was, “What is the subjective performance intensity of the sport you are currently participating in?” The latter question is an index of postoperative subjective athletic performance (PoSAP) [29]. The PoSAP index ranges from 0 to 100% and reflects the athlete’s performance level relative to their pre-ACL injury performance. Most post-ACLR patients with a PoSAP of 80% responded “Yes” to the dichotomous question, and that the dichotomous question alone tended to over evaluate their RTS status [29]. Therefore, in this study, the participants who answered “Yes” to the dichotomous question and > 80% for the PoSAP were included in the Yes-RTS (YRTS) group. The No-RTS (NRTS) group included those who met none or only one of these criteria.

### Statistical analysis

The normality of each variable’s distribution was determined by histogram and the Shapiro–Wilk normality test. Differences between the NRTS and YRTS groups were analysed using the chi-squared test, Fisher’s exact test, unpaired *t*-test, or Mann‒Whitney's U test. Additionally, effect sizes (chi-squared test = φ coefficient, Fisher’s exact test = Cramer’s V, t-test = Cohen’s d, Mann‒Whitney’s U test = r) were calculated for each variable.

The SLHD cut-off point (%body height) was set based on the minimum *p*-value approach [25]. The minimum *p*-value approach is a method for finding the optimal threshold influencing the outcome among continuous variables. First, the SLHD distribution (%body height) on the operated side was identified and the cut-off points classified as 60, 70, 80, 90, and 100% of body height, respectively. Next, a chi-square test was performed for each RTS status and the SLHD cut-off point, and the value with the highest chi-square value and lowest *p*-value was set as cut-off point [25]. A post-hoc power analysis was performed to assess the association between RTS status and the SLHD cut-off point using G*power software 3.1.9.4 [10].

The associations between SLHD performance and RTS status were examined using logistic regression analysis (forced entry method). First, the analysis was performed with the SLHD (%body height [cut-off value] and asymmetry [LSI]) as independent variables (Model 1) and then adjusted for variables with *p* < 0.10 and potentially confounding variables in the between-group comparisons (Model 2). Among the body composition variables, body weight with a larger effect size was entered as a covariate. The a priori α level was set at 0.05. Data were analysed using SPSS version 21.0 (IBM Corp, Armonk, NY, USA).

## Results

Seventy-three participants after ACLR were included in this study. Thirty (41%) and 43 (59%) participants were assigned to the NRTS and YRTS groups, respectively. Seven of the 51 participants (14%) who answered “Yes” to the dichotomous question were PoSAP ≤ 80% and three of the 47 participants (7%) who had a PoSAP > 80% answered "No" to the dichotomous question.

Demographic and functional variables are shown in Tables 1 and 2. The NRTS group had a significantly higher TSK score and a smaller Ext 180 LSI value and SLHD (%body weight) than the YRTS group.

**Table 2:** Strength and single-leg hop distance variables in participants

		RTS status			
	Total (*n* = 73)	YRTS (*n* = 43)	NRTS (*n* = 30)	*P*-value	Effect size
SLHD LSI, %	98.9 ± 6.6	99.6 ± 6.6	97.5 ± 6.5	0.182	0.32
SLHD on operated side (%body height)	78.5 ± 15.8	81.9 ± 13.2	73.5 ± 18.1	0.025	0.55
Ext 60 LSI (%)	91.1 ± 11.9	93.0 ± 11.6	89.1 ± 12.2	0.165	0.33
Ext 180 LSI (%)	90.1 ± 9.2	92.3 ± 8.3	87.0 ± 9.5	0.013	0.60
Flex 60 LSI (%)	92.0 ± 10.8	92.3 ± 11.6	92.1 ± 11.4	0.930	0.02
Flex 180 LSI (%)	92.0 ± 11.5	93.8 ± 11.7	89.5 ± 10.6	0.116	0.38

*ACLR* anterior cruciate ligament reconstruction, *RTS* return-to-sports, *YRTS* yes‒return-to-sports, *NRTS* no‒return-to-sports, *SLHD* single-leg hop distance, *LSI* limb symmetry index

Ext and Flex 60/180 represent the isokinetic knee extension and flexion torque at 60°/s or 180°/s

When the SLHD cut-off point (%body height) on the operated side was set at 70%, the *p*-value was the smallest, and the chi-square value largest (*p* = 0.004, chi-square value = 8.071) (Table 3). Post-hoc test results showed a power of 0.81 (effect size = 0.333, a = 0.05, sample size = 73, Df = 1). The distribution for cut-off points of 60% and 100% was small, so that chi-square values could not be calculated. Therefore, the SLHD cut-off point (%body height) on the operated side used for logistic regression analysis was set at 70%.

**Table 3:** Relationship between return-to-sports status and single-leg hop for distance on the operated side

		RTS status			
	SLHD on the operated side (% body height)	YRTS	NRTS	*P-value*	χ^2^ value
Cut-off 100%	≥ 100	6	3	-	-
	< 100	37	27		
Cut-off 90%	≥ 90	12	5	0.264	1.250
	< 90	31	25		
Cut-off 80%	≥ 80	23	11	0.156	2.010
	< 80	20	19		
Cut-off 70%	≥ 70	35	15	0.004	8.071
	< 70	8	15		
Cut-off 60%	≥ 60	42	22	-	-
	< 60	1	8		

*ACLR* anterior cruciate ligament reconstruction, *RTS* return-to-sports, *YRTS* yes‒return-to-sports, *NRTS* no‒return-to-sports, *SLHD* single-leg hop distance

The results of the logistic regression analysis are shown in Table 4. In Model 1, the SLHD on the operated side (%body height), using a cut-off point of 70%, was associated with RTS status (*p* = 0.013, odds ratio [95 confidence interval] = 3.980 [1.338‒11.840]. In Model 2, the SLHD (%body height) cut-off point 70% (*p* = 0.013, odds ratio = 9.602 [1.598‒57.689]) and TSK (*p* = 0.014, odds ratio = 1.173 [1.033‒1.331]) were factors associated with RTS status.

**Table 4:** Logistic regression analysis to determine the association between single-leg hop performance and return-to-sports status

Logistic regression analysis	*P*-value	Odds ratio	95% Confidence interval	
Independent Variable			Lower	Upper
Model 1				
The LSI of the SLHD	0.556	0.975	0.896	1.061
SLHD on the operated side (%body height) cut-off 70%	0.013	3.980	1.338	11.840
Model χ^2^ test, *P* = 0.015; Hosmer‒Lemeshow test, *P* = 0.248.				
Percentage of correct classification, 68.5%				
Model 2				
Age	0.390	1.054	0.935	1.188
Sex	0.300	3.022	0.374	24.420
Body weight	0.725	1.011	0.950	1.077
Postoperative months	0.557	0.990	0.957	1.024
TSK	0.014	1.173	1.033	1.331
SLHD on the operated side (%body height) cut-off 70%	0.013	9.602	1.598	57.689
Ext 180 LSI	0.244	0.958	0.890	1.030
Model χ^2^ test, *P* < 0.001; Hosmer‒Lemeshow test, *P* = 0.390.				
Percentage of correct classification, 78.1%				

*LSI* limb symmetry index, *SLHD* single-leg hop distance, *TSK* Tampa scale for kinesiophobia

Ext 180 represents the isokinetic knee extension torque at 180°/s

## Discussion

The main finding of this study was that despite a good LSI improvement in SLHD, an SLHD % body height < 70% on the operative side was negatively associated with RTS status. These results support our hypothesis, and that the SLHD on the operated side (% body height) is associated with RTS status.

Among the participants in this study, 59% achieved RTS at the same level of competition as before their ACL injury. This result is comparable to that of a previous meta-analysis [3] that investigated the RTS rate (63%, 95% CI 54‒71%).

In the present study, a cut-off point of 70% of body height for the SLHD on the operated side was negatively associated with RTS status. No previous report has established a cut-off point for RTS status and the SLHD on the operated side in post-ACLR patients. In a cohort study examining the association between pre-season functional testing and in-season lower extremity injury in healthy female college athletes, injury rates were increased in those with an SLHD < 64‒70% body height [8, 9]. Although the subjects and outcomes of that study differed from those of the present study, the results of the present study are considered to provide a reasonable reference value for setting a 70% target SLHD % body height in post-ACLR patients.

In patients with an ACL injury, or after reconstructive surgery, the SLHD has been positively correlated with functional variables, such as knee extensor strength and hip external rotator strength [13, 18, 32]. Confidence in the knee and knee pain during hopping was associated with the SLHD on the operated side in patients one year after ACLR [14]. These findings suggest that the SLHD is a comprehensive performance test that includes muscle strength for jumping further, and knee pain, and confidence during hopping. The present study provides new evidence for the need to focus on the SLHD on the operated side (%body height) in addition to the LSI for RTS after ACLR.

In the present study, the LSI of isokinetic extension torque (180°/sec) and the TSK score were significantly related to RTS status. Kinesiophobia and knee extension muscle weakness on the operative side were important variables related to RTS status [5, 20, 22, 23]. In particular, TSK was also a significant variable in logistic regression analysis. Kinesiophobia (fear of pain and re-injury) was found to be a major factor inhibiting RTS in post-ACLR patients with good improvement in physical function [3], and the present study results support results of previous reports.

The LSI of the SLHD among the participants of this study was 97.5% and 99.6% for the NRTS and YRTS groups, respectively, with no significant difference between the groups (Cohen’s d = 0.32). A previous study [35] showed a statistically significant association between RTS status at minimum two years after ACLR and the LSI of the SLHD (including triple crossover hop for distance) at 12 months after ACLR, but the effect size calculated from that approach was small (NRTS: 95 ± 11%, YRTS: 98 ± 9%, Cohen’s d = 0.31). In another previous study, the association between LSI of SLHD at 6 months after ACLR and RTS status at 12 months after ACLR was statistically significant, and the calculated effect size large (NRTS: 77.9 ± 15.2%, YRTS: 90.6 ± 8.9%, Cohen’s d = 1.23) [17]. Thus, the LSI of SLHD has different characteristics depending on the time of measurement.

The SLHD is frequently used in post-ACLR rehabilitation for exercise and functional assessment because of its high measurement reproducibility and ease of use [1]. In particular, the SLHD is a standard measure for determining improvements in lower extremity function and RTS in facilities without isokinetic dynamometry. In post-ACLR rehabilitation, improving the LSI of the SLHD to 85‒90% or higher is one of the functional criteria for allowing sports participation [33]. Considering the results of this study, it may be important to plan rehabilitation and training with the goal of achieving an SLHD > 70% body weight, even after the LSI of the SLHD is > 90%, in order to achieve the same level of competition as before the injury.

There are several limitations to this study. This was a cross-sectional study, and the causal relationship between variables unknown. The duration since ACLR in the participants in this study was approximately 13.5 (IQR 13.0) months, which is a wide range. This duration was set with reference to reports of residual functional decline after more than 2 years post-ACLR [27, 30]. Only two of the participants in this study had an LSI of the SLHD < 90%; thus, this was a population with a good improvement in asymmetry. It is therefore unclear whether the results of this study can be adapted to cases of insufficient improvement of SLHD asymmetry. In this study, only distance was measured in SLHD. A recent review showed that even with satisfactory LSI improvement in SLHD, neuromuscular and biomechanical deficits are observed on the operated side when it is compared to the unoperated side [19]. These aspects could not be analysed in this study. We included only successful trials of SLHD in the analysis. Unsuccessful trials may show different neuromuscular characteristics than those in successful trials [37]; hence, it is necessary to include the proportion of unsuccessful trials. Future studies should be conducted to include neuromuscular and biomechanical variables and unsuccessful trials. Finally, in this study, the cut-off point was set at an SHLD of 70% body height, but this point may vary depending on sex and level of competition. The power in the post-hoc analysis of the association between a 70% SLHD (% body height) cut-off point and RTS status was acceptable (β = 0.81), but the lack of a sample size big enough prevented analysis at 60% and 100%. Future subgroup analyses of larger sample size should be undertaken to resolve these issues.

## Conclusion

An SLHD % body height < 70% on the operative side was negatively associated with RTS status. Our results suggest that even after improvement in the LSI of the SLHD, planning rehabilitation and training with the goal of achieving an SLHD > 70% body height may be important to support RTS after ACLR.

## Abbreviations

ACL: Anterior cruciate ligament
ACLR: Anterior cruciate ligament reconstruction
LSI: Limb symmetry index
NRTS: No‒return-to-sports
PoSAP: Postoperative subjective athletic performance
RTS: Return-to-sports
SLHD: Single-leg hop distance
TSK: Tampa Scale of Kinesiophobia
YRTS: Yes‒return-to-sports

## References

[1] Abrams GD, Harris JD, Gupta AK, McCormick FM, Bush-Joseph CA, Verma NN (2014). Functional performance testing after anterior cruciate ligament reconstruction: a systematic review. Orthop J Sports Med.

[2] Ardern CL, Taylor NF, Feller JA, Whitehead TS, Webster KE (2015). Sports participation 2 years after anterior cruciate ligament reconstruction in athletes who had not returned to sport at 1 year: a prospective follow-up of physical function and psychological factors in 122 athletes. Am J Sports Med.

[3] Ardern CL, Webster KE, Taylor NF, Feller JA (2011). Return to sport following anterior cruciate ligament reconstruction surgery: a systematic review and meta-analysis of the state of play. Br J Sports Med.

[4] Ardern CL, Webster KE, Taylor NF, Feller JA (2011). Return to the preinjury level of competitive sport after anterior cruciate ligament reconstruction surgery: two-thirds of patients have not returned by 12 months after surgery. Am J Sports Med.

[5] Ardern CL, Österberg A, Tagesson S, Gauffin H, Webster KE, Kvist J (2014). The impact of psychological readiness to return to sport and recreational activities after anterior cruciate ligament reconstruction. Br J Sports Med.

[6] Barfod KW, Feller JA, Hartwig T, Devitt BM, Webster KE (2019). Knee extensor strength and hop test performance following anterior cruciate ligament reconstruction. Knee.

[7] Brosky JA, Nitz AJ, Malone TR, Caborn DN, Rayens MK (1999). Intrarater reliability of selected clinical outcome measures following anterior cruciate ligament reconstruction. J Orthop Sports Phys Ther.

[8] Brumitt J, Heiderscheit BC, Manske RC, Niemuth PE, Mattocks A, Rauh MJ (2018). Preseason functional test scores are associated with future sports injury in female collegiate athletes. J Strength Cond Res.

[9] Brumitt J, Mattocks A, Loew J, Lentz P (2019). Preseason functional performance test measures are associated with injury in female college volleyball players. J Sport Rehabil.

[10] Faul F, Erdfelder E, Lang AG, Buchner A (2007). G*Power 3: a flexible statistical power analysis program for the social, behavioral, and biomedical sciences. Behav Res Methods.

[11] Feucht MJ, Cotic M, Saier T, Minzlaff P, Plath JE, Imhoff AB (2016). Patient expectations of primary and revision anterior cruciate ligament reconstruction. Knee Surg Sports Traumatol Arthrosc.

[12] Fältström A, Hägglund M, Kvist J (2013). Patient-reported knee function, quality of life, and activity level after bilateral anterior cruciate ligament injuries. Am J Sports Med.

[13] Greenberger HB, Paterno MV (1995). Relationship of knee extensor strength and hopping test performance in the assessment of lower extremity function. J Orthop Sports Phys Ther.

[14] Hart HF, Culvenor AG, Guermazi A, Crossley KM (2020). Worse knee confidence, fear of movement, psychological readiness to return-to-sport and pain are associated with worse function after ACL reconstruction. Phys Ther Sport.

[15] Kikuchi N, Matsudaira K, Sawada T, Oka H (2015). Psychometric properties of the Japanese version of the Tampa Scale for Kinesiophobia (TSK-J) in patients with whiplash neck injury pain and/or low back pain. J Orthop Sci.

[16] Kitaguchi T, Tanaka Y, Takeshita S, Akizaki K, Takao R, Kinugasa K (2020). Preoperative quadriceps strength as a predictor of return to sports after anterior cruciate ligament reconstruction in competitive athletes. Phys Ther Sport.

[17] Kitaguchi T, Tanaka Y, Takeshita S, Tsujimoto N, Kita K, Amano H (2020). Importance of functional performance and psychological readiness for return to preinjury level of sports 1 year after ACL reconstruction in competitive athletes. Knee Surg Sports Traumatol Arthrosc.

[18] Kline PW, Burnham J, Yonz M, Johnson D, Ireland ML, Noehren B (2018). Hip external rotation strength predicts hop performance after anterior cruciate ligament reconstruction. Knee Surg Sports Traumatol Arthrosc.

[19] Kotsifaki A, Korakakis V, Whiteley R, Van Rossom S, Jonkers I (2020). Measuring only hop distance during single leg hop testing is insufficient to detect deficits in knee function after ACL reconstruction: a systematic review and meta-analysis. Br J Sports Med.

[20] Kvist J, Ek A, Sporrstedt K, Good L (2005). Fear of re-injury: a hindrance for returning to sports after anterior cruciate ligament reconstruction. Knee Surg Sports Traumatol Arthrosc.

[21] Langford JL, Webster KE, Feller JA (2009). A prospective longitudinal study to assess psychological changes following anterior cruciate ligament reconstruction surgery. Br J Sports Med.

[22] Lentz TA, Zeppieri G, George SZ, Tillman SM, Moser MW, Farmer KW (2015). Comparison of physical impairment, functional, and psychosocial measures based on fear of reinjury/lack of confidence and return-to-sport status after ACL reconstruction. Am J Sports Med.

[23] Lentz TA, Zeppieri G, Tillman SM, Indelicato PA, Moser MW, George SZ (2012). Return to preinjury sports participation following anterior cruciate ligament reconstruction: contributions of demographic, knee impairment, and self-report measures. J Orthop Sports Phys Ther.

[24] Lepley LK, Palmieri-Smith RM (2015). Quadriceps strength, muscle activation failure, and patient-reported function at the time of return to activity in patients following anterior cruciate ligament reconstruction: a cross-sectional study. J Orthop Sports Phys Ther.

[25] Mazumdar M, Glassman JR (2000). Categorizing a prognostic variable: review of methods, code for easy implementation and applications to decision-making about cancer treatments. Stat Med.

[26] Nagai T, Schilaty ND, Laskowski ER, Hewett TE (2020). Hop tests can result in higher limb symmetry index values than isokinetic strength and leg press tests in patients following ACL reconstruction. Knee Surg Sports Traumatol Arthrosc.

[27] Nagelli CV, Hewett TE (2017). Should return to sport be delayed until 2 years after anterior cruciate ligament reconstruction? biological and functional considerations. Sports Med.

[28] Nwachukwu BU, Adjei J, Rauck RC, Chahla J, Okoroha KR, Verma NN (2019). How much do psychological factors affect lack of return to play after anterior cruciate ligament reconstruction? A systematic review. Orthop J Sports Med.

[29] Ohji S, Aizawa J, Hirohata K, Ohmi T, Koga H, Okawa A (2020). The gap between subjective return to sports and subjective athletic performance intensity after anterior cruciate ligament reconstruction. Orthop J Sports Med.

[30] Palmieri-Smith RM, Thomas AC, Wojtys EM (2008). Maximizing quadriceps strength after ACL reconstruction. Clin Sports Med.

[31] Patterson BE, Crossley KM, Perraton LG, Kumar AS, King MG, Heerey JJ (2020). Limb symmetry index on a functional test battery improves between one and five years after anterior cruciate ligament reconstruction, primarily due to worsening contralateral limb function. Phys Ther Sport.

[32] Pua YH, Ong PH, Ho JY, Bryant AL,  WebsterClark KERA (2015). Associations of isokinetic knee steadiness with hop performance in patients with ACL deficiency. Knee Surg Sports Traumatol Arthrosc.

[33] Thomeé R, Kaplan Y, Kvist J, Myklebust G, Risberg MA, Theisen D (2011). Muscle strength and hop performance criteria prior to return to sports after ACL reconstruction. Knee Surg Sports Traumatol Arthrosc.

[34] Webster KE, Feller JA (2018). Return to level I sports after anterior cruciate ligament reconstruction: evaluation of age, sex, and readiness to return criteria. Orthop J Sports Med.

[35] Webster KE, McPherson AL, Hewett TE, Feller JA (2019). Factors associated with a return to preinjury level of sport performance after anterior cruciate ligament reconstruction surgery. Am J Sports Med.

[36] Wellsandt E, Failla MJ, Snyder-Mackler L (2017). Limb symmetry indexes can overestimate knee function after anterior cruciate ligament injury. J Orthop Sports Phys Ther.

[37] Wikstrom EA, Tillman MD, Schenker S, Borsa PA (2008). Failed jump landing trials: deficits in neuromuscular control. Scand J Med Sci Sports.

[38] Woby SR, Roach NK, Urmston M, Watson PJ (2005). Psychometric properties of the TSK-11: a shortened version of the Tampa Scale for Kinesiophobia. Pain.

[39] Xergia SA, McClelland JA, Kvist J, Vasiliadis HS, Georgoulis AD (2011). The influence of graft choice on isokinetic muscle strength 4–24 months after anterior cruciate ligament reconstruction. Knee Surg Sports Traumatol Arthrosc.

